# Re-sequencing of the casein genes in Swedish Red cattle giving milk with diverse protein profiles and extreme rennet coagulation properties

**DOI:** 10.3168/jdsc.2023-0412

**Published:** 2024-02-29

**Authors:** Frida Lewerentz, Tytti K. Vanhala, Lene Buhelt Johansen, Marie Paulsson, Maria Glantz, Dirk-Jan de Koning

**Affiliations:** 1 Department of Process and Life Science Engineering, Division of Food and Pharma, Lund University, SE-221 00 Lund, Sweden; 2 Department of Animal Breeding and Genetics, Swedish University of Agricultural Sciences, SE-750 07 Uppsala, Sweden; 3 Arla Foods Innovation Center, DK-8200 Aarhus, Denmark

## Abstract

Impaired rennet coagulation properties in milk could lead to prolonged processing times and production losses. Heritability for milk coagulation has previously been estimated to be 0.28 to 0.45, indicating that genetic selection can be used to manipulate this trait. The CN proteins are expressed by the genes *CSN1S1, CSN2, CSN1S2*, and *CSN3* and are located on bovine chromosome 6. To better understand the effect of genetic variation in the CN genes on milk coagulation, blood and milk samples from 30 Swedish Red Dairy Cattle (RDC) with divergent coagulation properties were investigated. DNA from the 30 cows was sequenced for the CN genes to determine the theoretical AA sequence and to look for genetic variation in the untranslated regions. The aim is to confirm the protein genetic variants previously reported, while searching for additional genetic variation in the CN genes of 30 RDC. We observed genetic variation in 116 SNPs in the known CN genes where 10% of the SNPs are exon variants and the remaining 90% are intron variants. A total of 2.5% of the SNPs are found in the 5′- or 3′-untranslated region (UTR) regions of the exons; 2% are synonymous variants and 6% are missense variants that concurred with the known protein variants for *CSN1S1, CSN2*, and *CSN3*. Furthermore, 6% of the SNPs are splice polypyrimidine tract intron variants. The 2 genetic variants in the 5′- and 3′-UTR in *CSN1S1* and *CSN3* are found with protein variants *CSN1S1C* and *CSN3B*. Because both UTR variants are associated with gain and loss of micro RNA and transcription factors, this could explain differences in expression of the genetic protein variants. Preliminary chi-squared analysis and comparison with previous GWAS studies showed potential connections between the identified SNPs and coagulation properties of milk. By advancing the knowledge of the connection between the DNA sequence and the functional properties of the CN proteins, we hope to learn more about the cheese coagulation properties of milk from RDC.

## Highlights

• Sequenced casein genes in 30 Swedish Red dairy cows confirmed protein variants.
• We found 121 genetic variants; 12 of which have not been previously described.
• Three genetic variants were found in the untranslated regions of exons in 3 caseins.
• Genetic variants could be potential markers for protein composition and coagulation.

## Graphical Abstract

**Figure:**
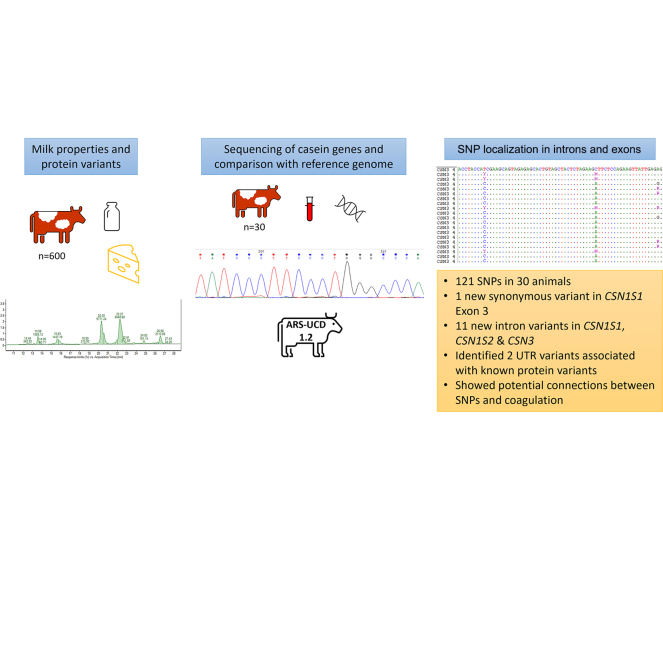
**Summary:** Impaired rennet coagulation properties in milk could lead to prolonged processing times and production losses. Heritability for impaired coagulation has previously been estimated to be 0.28 to 0.45, indicating that genetic selection can be used to manipulate this trait. To better understand the effect of genetic variation in the casein genes on milk coagulation, blood samples from 30 cows with divergent coagulation properties were sequenced. We found 121 genetic variants, including 12 that have not been previously described and 1 synonymous variant in CSN1S1. Three genetic variants were found in the untranslated regions of exons in 3 caseins. Genetic variants could be potential markers for protein composition and coagulation.

Rennet coagulation of milk is crucial to cheese production and milk with impaired coagulation could lead to prolonged processing times and production losses. In Swedish Red Dairy Cattle (**RDC**) up to 37% of the cows produce poor or noncoagulating (**NC**) milk, and the heritability is 0.28 to 0.45 for NC milk (Gustavsson et al., 2014; Duchemin et al., 2020). Both Gustavsson et al. (2014) and Duchemin et al. (2020) showed strong phenotypic and genetic correlations between the detailed CN composition and the coagulation traits. There are 4 different CN in bovine milk, α_S1_-, β-, α_S2_-, and κ-CN, with the corresponding genes *CSN1S1, CSN2, CSN1S2*, and *CSN3* all closely located on bovine chromosome 6. There are several genetic variants of the CN and their prevalence varies among cows and breeds (Caroli et al., 2009). Genetic protein variants can be identified by protein analysis relying on electric charge, molecular weight, or isoelectric point. However, as some genetic protein variants share the same mass or isoelectric point, such as β-CN F and G, genetic sequencing is needed for determination. Furthermore, there are exon variations in the CN genes not affecting the mature protein per se, causing synonymous variants (differences in DNA sequence but not in the translated AA sequence), SNPs in the untranslated regions (**UTR**) of the exons that can affect protein expression, and SNPs in splice regions that can lead to splice variants of the proteins (Boeva, 2016). The UTR are part of the mRNA but occur before the start codon signaling for translation of mRNA to protein or occur after the end codon. These regions can affect mRNA stability, mRNA localization, and translational efficiency (Barrett et al., 2013). Nilsson et al. (2020) studied different milk protein variants and their post-translational modifications in relation to rennet coagulation using liquid chromatography high-resolution mass spectrometry (**LC-HRMS**). Protein variants β-CN A2 and κ-CN A were more common in NC milk and β-CN A1 and κ-CN B were more common in coagulating milk. These effects were believed to be indirect as the *CSN2* genotypes influence relative concentrations of α_S2_- and κ-CN and the *CSN3* genotypes affect relative concentrations of α_S1_- and κ-CN. Meanwhile, Lewerentz et al. (2023) found that relative concentrations of κ-CN explained much of the variation in rennet coagulation properties. To better understand the effect of genetic variation in the CN genes on milk coagulation, blood and milk samples from 30 RDC with divergent coagulation properties were chosen from a previous observational study on 600 RDC cows (Duchemin et al., 2020). The DNA was sequenced for the CN genes to determine the theoretical AA sequence and to look for genetic variation in introns and untranslated exon regions. The aim of this study was to confirm the protein-based genetic variants previously reported, while also searching for additional genetic variation, specifically synonymous variants in the exons of 30 RDC. Furthermore, we hypothesized that the discovery of SNPs in the UTR of exons and introns could have an effect on protein expression.

Of the 600 RDC cows included in our previous observational study in December 2015 to April 2017 (Duchemin et al., 2020), 30 cows were selected based on their milk properties with regard to rennet coagulation properties, protein genetic variants, and post-translational modifications (Nilsson et al., 2020). The selected cows came from 26 of the 31 conventional farms in the South of Sweden included in the previous study (Duchemin et al., 2020) where 2 cows/farm were selected in 4 farms and one cow/farm was selected in the rest of the farms. All 30 cows had different sires and dams. For the 30 cows, milk samples were collected at d 194 ± 78 (average ± SD) in the lactation cycle and in parity 2.2 ± 1.6, which is in the range of what was found in the full dataset (Nilsson et al., 2020). Of the cows investigated in this study, 13 cows gave milk that did not coagulate within 40 min after rennet addition (rennet coagulation time [**RCT**] >40 min) and 17 cows gave milk with intermediate to good coagulation properties defined by gel strength (G′_max_ = 412 ± 226 Pa) and RCT (10 ± 1.1 min). Furthermore, previous information on genetic protein variants and post-translational modification variants in Nilsson et al. (2020) was used to choose a subset with a large genetic variation regarding protein. Hence, the allele frequency of the more rare genetic protein variants is somewhat higher in the subset than in the full sample set of 600 cows from Nilsson et al. (2020). The methods for measuring rennet coagulation and protein composition can be found in Nilsson et al. (2020). The DNA from the 30 cows was available in the bio-bank at the Swedish University of Agricultural Sciences, Uppsala, and was sequenced using primer sequences from the exons of all 4 CN genes according to Gallinat et al. (2013) resulting in 34 sequencing fragments. Sanger sequencing was done using the BigDye Direct Cycle Sequencing kit (Applied Biosystems, Thermo Fisher Scientific, Waltham, MA). We followed the sequencing protocol as described in the BigDye instruction manual. Purification of the sequencing reactions was performed with BigDye XTerminator kit (Applied Biosystems, Thermo Fisher Scientific). The sequencing reactions were then run in the Genetic Analyzer 3500xL (Applied Biosystems, Thermo Fisher Scientific). Sequence data were checked and edited manually using the free trace viewer Chromas 2.5 (Technelysium Pty Ltd., Brisbane, QLD, Australia) and BioEdit 7.2. (Hall, 1999). This was also the step where the variant calling was performed through manual inspection of the Sanger sequences. The obtained sequences were aligned across all animals and the reference sequence in BioEdit. This allowed the identification of any sequence variants among the animals in relation to the reference sequence. All exons in the CN genes that are translated to proteins are covered in the sequencing. Introns directly preceding and following translated exons are covered giving an average of 37% intron coverage per gene. The sequences were aligned and compared with the latest version of the bovine genome sequence ARS-UCD1.2 and the reference genes ENSBTAG00000007695 (*CSN1S1*), ENSBTAG00000005005 (*CSN1S2*), ENSBTAG00000002632 (*CSN2*), and ENSBTAG00000039787 (*CSN3*). The positions of the detected SNPs were checked against the Ensembl genome database (release 109, Feb. 2023; https://www.ensembl.org/Bos_taurus/) to determine if they had been previously described and find the correct reference SNP number (**rsID**). Chi-squared analysis was performed using Minitab 20 (Minitab Ltd.) to investigate if there were any associations between the allele distribution of the newly identified SNPs and the 2 coagulation groups.

The sequencing of the casein genes in the 30 individual RDC yielded 116 SNPs where each SNP was present in at least one of the individuals. Furthermore, 5 SNPs were identified in which all 30 individuals differed from the reference sequence ARS-UCD1.2. The majority of the SNPs, 84%, are located in the introns on positions without any expected consequence to transcription or translation (Figure 1). Furthermore, 6% of the SNPs can be classed as splice polypyrimidine tract variants, which are SNPs that fall in the polypyrimidine tract at the 3′ end of intron between 17 and 3 bases from the end (SO:0002169; Eilbeck et al., 2005). The rest of the SNPs are situated in the exons where 2.5% of the SNPs present in the subset can be found in the 5′- or 3′-UTR of the exons, 1.7% of the SNPs are synonymous variants, and 6% of the SNPs are missense variants concurring with the known protein variants for *CSN1S1, CSN2*, and *CSN3*.

**Figure 1:**
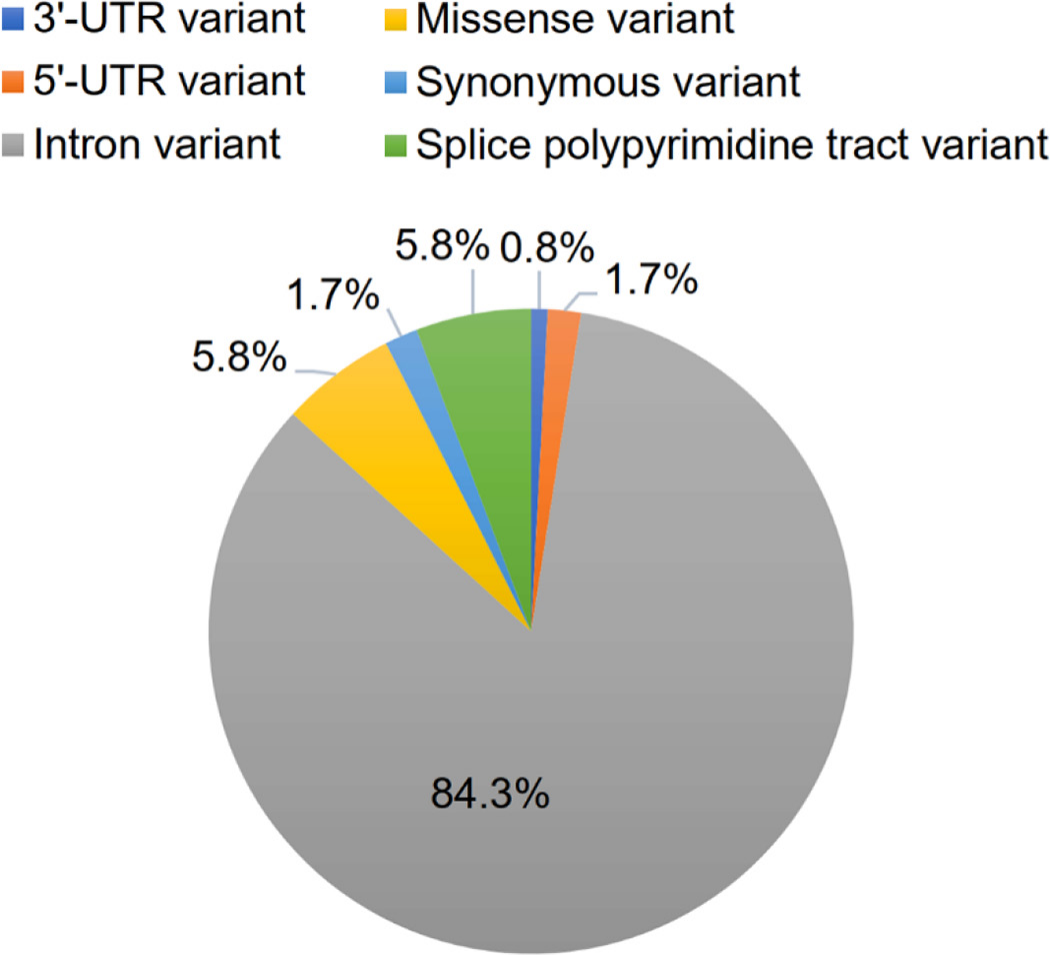
Distribution of the different DNA variant types in the 121 SNPs found in the 30 RDC cows.

The SNPs found in exons are presented in Table 1. The variants *CSN1S1*^C^, *CSN2*^B^, and *CSN2*^F^ had an allele frequency of 0.03, 0.02, and 0.02, respectively, in the subset, whereas the frequency was substantially lower in the collected sample set where the protein variants were determined using the protein phenotype (Nilsson et al., 2020). The allele frequency of the protein variants of the rest of *CSN1S1* and *CSN2* as well as all protein variants of *CSN3* was very close to that of the full sample set presented in Nilsson et al. (2020). As the 30 animals used in this study were chosen with regard to extreme coagulation properties as well as achieving a large variation in genetic protein variants, it is likely that the more uncommon genetic protein variants are overrepresented compared with the RDC population.

**Table 1:** Description of the genetic variants found in exons of the 4 casein genes *CSN1S1, CSN1S2, CSN2*, and *CSN3*^1^

SNP						Gene transcript	
Gene SNP ID^2^	BTA:bp^3^	Exon	Allele (ref/alt)^4^	AF^5^ (n = 30)	AF in NC^5^ (n = 13)	AA exchange and position^6^	Genetic protein variant
*CSN1S1*							
rs110899610	6:85413195	2	C/T	0.97/0.03	0.96/0.04	—7	[B]/[C]
ss9732325453	6:85415215	3	T/C	0.98/0.02	0.96/0.04	His4His^8^	—
rs43703010	6:85427427	17	A/G	0.97/0.03	0.96/0.04	Glu192Gly	[B]/[C]
*CSN1S2*							
rs109900747	6:85531897	2	T/C	0.47/0.53	0.31/0.69*	—7	—
*CSN2*							
rs43703011	6:85451298	7	A/C	0.42/0.58	0.27/0.73*	His67Pro	[A1, F]/[A2, B]
rs43703013	6:85451132	7	C/G	0.98/0.02	1.00/0.00	Ser122Arg	[A1, A2, F]/[B]
rs433954503	6:85451043	7	C/T	0.98/0.02	1.00/0.00	Pro152Leu	[A1, A2, B]/[F]
*CSN3*							
rs43703015	6:85656736	4	C/T	0.80/0.20	0.81/0.19	Thr136Ile	[A, E]/[B]
rs43703016	6:85656772	4	A/C	0.80/0.20	0.81/0.19	Thr148Ala	[A, E]/[B]
rs43703017	6:85656792	4	A/G	0.85/0.15	0.96/0.04*	Ser155Gly	[A, B]/[E]
rs110014544	6:85656833	4	A/G	0.80/0.20	0.81/0.19	Ala168Ala^8^	[A, E]/[B]
rs109787476	6:85656841	4	A/T	0.80/0.20	0.81/0.19	—9	[A, E]/[B]

1 SNPs are identified with rsID/ssID, genome position, exon, and allele frequency (AF) in 30 animals, allele frequency of the 13 animals producing noncoagulating (NC) milk together with the resulting protein variants.

2 Variant ID: rs-numbers retrieved from the Ensembl database; ss-accession numbers of new variants identiﬁed from the present sequencing assigned by the European Variation Archive.

3 bp = SNP position according to the ARS-UCD1.2 genome.

4 ref/alt = reference/alternative allele.

5 Allele frequency (AF) in the reference/alternative allele.

6 Amino acid position in mature protein.

7 5′-untranslated (UTR) variant.

8 Synonymous variant with the same AA but different alleles.

9 3′-untranslated (UTR) variant.

* Significant association between coagulation trait and AF (*P* < 0.05).

In exon 17 of *CSN1S1* we confirmed the presence of, for RDC, the rare variant *CSN1S1*^C^ in the 2 animals that was previously observed in the protein analyses (Nilsson et al., 2020). The remaining animals were homozygous for *CSN1S1*^B^. The 2 animals that were heterozygous for *CSN1S1*^C^ also showed a mutation in the 5′ UTR in exon 2 in *CSN1S1*, located 1 bp upstream the start of the translated sequence (rs110899610T>C, Table 1). This genetic variant was also found in a study on Beninese cattle breeds by Vanvanhossou et al. (2021). *CSN1S1*^C^ was present in the dataset, but the authors did not present any data regarding if there is any linkage between rs110899610C and *CSN1S1*^C^. Vanvanhossou et al. (2021) performed an analysis to investigate SNPs in binding sites of transcription factors (**TF**) or microRNA (**miRNA**). They found that the change from C to T in rs110899610 could cause a loss of binding of miRNAs bta-miR-2420 and bta-miR-453 as well as a gain of binding of bta-miR-376a. Because miRNA can affect gene expression, the mutation in the 5′ UTR region of the *CSN1S1* could affect the composition of produced milk (Boeva, 2016). Interestingly, a synonymous variant was found on exon 3 in *CSN1S1*, position 6:85415215 (ss9732325453, Table 1), changing the DNA sequence from T to C in one of the 30 animals. In both genetic variants the AA sequence remains the same with His on position 4 in the mature protein. This synonymous variant is not present in the Ensembl or NCBI databases and has, to our knowledge, not previously been reported.

All animals were found to be homozygote for the *CSN1S2*^B^, which was consistent with the protein analyses (Nilsson et al., 2020). There was one SNP (rs109900747T>C, Table 1) found in the 5′-UTR region on exon 2 in *CSN1S2*, positioned 5 bp upstream of the translated sequence. This genetic variant was also found in the study by Vanvanhossou et al. (2021) and the authors suggest that a change from rs109900747T to C results in a gain of binding bta-miR-452 and loss of binding of several TF. There is considerable variation among the animals in this study regarding this SNP as 47% of the animals are heterozygous, 23% are homozygous for the T allele, and 30% are homozygous for the C allele (Table 1).

As in the full sample set (Nilsson et al., 2020), *CSN2*^A1^ and *CSN2*^A2^ were the most common β-CN protein variants in the subset of RDC with a frequency of 0.40 and 0.56, respectively (Table 1). The DNA sequencing confirmed the presence of, in RDC, the rare protein variants *CSN2*^B^ and *CSN2*^F^ caused by SNPs rs43703013C>G and rs433954503C>T, located on exon 7. In *CSN2*^B^, rs43703013G results in an Arg on AA position 122 and in *CSN2*^F^, rs433954503T results in a Leu on position 152 in the mature protein. rs43703011A results in a His in AA position 67 in the mature protein in variants *CSN2*^F^ and *CSN2*^A1^, whereas rs43703011C result in a Pro in the same position in variants *CSN2*^B^ and *CSN2*^A2^. The protein variants *CSN2*^A1^ and *CSN2*^A2^ have especially caught attention in recent research. In a systematic review of randomized controlled trials, Daniloski et al. (2021) conclude that milk with β-CN protein variant A2 can have a positive effect on human health compared with the A1 variant.

On exon 4 in *CSN3*, the 3 SNPs rs43703015C>T, rs43703016A>C, and rs43703017A>G describe protein variants *CSN3*^A^, *CSN3*^B^, and *CSN3*^E^. CSN^A^ and CSN^E^ have a Thr on AA positions 157 and 159 in the mature protein, whereas CSN^B^ has Ile and Ala in the same positions. CSN^E^ is separated from CSN^A^ with a Gly instead of Ser on position 155 in the mature protein. There is also a synonymous variant, rs110014544A>G, and a 3′ UTR variant, rs109787476A>T, on exon 4, *CSN3*. The G and T alleles of these 2 SNPs concur with *CSN3*^B^. Vanvanhossou et al. (2021) suggested that a change from T to A in rs109787476 causes loss in binding of miRNA bta-miR-496 and TF ZNF274 as well as a gain in binding of bta-miR-2284w. As reviewed by Hewa Nadugala et al. (2022), *CSN3*^B^ has been associated with higher total CN but also higher κ-CN contents in milk. The loss and gain of miRNA and TF could theoretically be part of an explanation for these superior qualities associated with *CSN3*^B^.

As presented in Figure 1, there is also quite a large amount of genetic variation in the introns of the CN genes. Twelve new SNPs and indels were found with variation in the dataset. Furthermore, 2 indels were the same in all 30 animals but were different from the reference gene. All in all, 109 intronic genetic variants were found. In Table 2, 9 of the new SNPs and indels are presented with their submitted SNP number (**ssID**) assigned by the European Variation Archive together with some known SNPs and indels, which were of specific interest in relation to intron position as well as potential correlation with protein composition or rennet coagulation. In total, half of the genetic variants identified in introns were found at a very low frequency (0.02–0.08) in the 30 animals and some are presented in Table 2. The rest of the SNPs and indels presented in Table 2 showed more variation among the animals with allele frequencies between 0.22 and 0.67.

**Table 2:** Genome positions for SNPs found in introns of the 4 casein genes *CSN1S1, CSN1S2, CSN2*, and *CSN3* including allele frequencies (AF) in 30 animals and in the 13 animals producing noncoagulating (NC) milk^1^

Gene SNP ID^2^	BTA:bp^3^	Allele (ref/alt)^4^	Intron	AF^5^ (n = 30)	AF in NC^5^ (n = 13)	Variant consequence type
*CSN1S1*						
rs137349211	6:85424552	C/T	13	0.77/0.23	0.89/0.11	Splice pol. tract
rs110440863	6:85422786	T/C	12	0.42/0.58	0.31/0.69	Intron
rs109193501	6:85424759	A/G	14	0.92/0.08	0.92/0.08	Intron
ss9732325462	6:85413132	TT/T^6^	1	0.97/0.03	0.96/0.04	Intron
ss9732325456	6:85415203	TT/T^6^	2	0.33/0.67	0.23/0.77	Splice pol. tract
ss9732325455	6:85416195	CTTC/C^6^	4	0.97/0.03	0.96/0.04	Intron
ss9732325458	6:85425555	TT/T^6^	13	0.97/0.03	0.96/0.04	Splice pol. tract
ss9732325457	6:85425495	TTT/T^6^	14	0.97/0.03	0.96/0.04	Splice pol. tract
*CSN1S2*						
rs110808655	6:85534747	T/C	5	0.62/0.38	0.77/0.23*	Intron
rs379988962	6:85535109	G/T	5	0.95/0.05	0.96/0.04	Splice pol. tract
rs110122319	6:85539636	T/C	12	0.60/0.40	0.73/0.27	Intron
rs133836703	6:85539717	C/T	12	0.52/0.48	0.65/0.35*	Intron
ss9732325449	6:85541964	T/TT^7^	15	0.60/0.40	0.73/0.27	Intron
ss9732325454	6:85542736	T/TT^7^	15	0.52/0.48	0.65/0.35	Intron
*CSN2*						
rs110466181	6:85450710	G/A	7	0.52/0.48	0.35/0.65*	Intron
rs110291532	6:85454960	A/AT^7^	2	0.40/0.60	0.23/0.77*	Intron
*CSN3*						
rs136419748	6:85648236	T/A	1	0.73/0.27	0.88/0.12*	Intron
ss9732325461	6:85656352	T/TT^7^	3	0.78/0.22	0.88/0.12	Intron

1 The SNPs presented in this table are either new SNPs with ssID, classed as splice polypyrimidine (pol.) tract variants, or SNPs of specific interest discussed in the present paper with rsID.

2 Variant ID: rs-numbers retrieved from the Ensembl database; ss-accession numbers of new variants identiﬁed from the present sequencing assigned by the European Variation Archive.

3 bp = SNP position according to the ARS-UCD1.2 genome.

4 ref/alt = reference/alternative allele.

5 Allele frequency (AF) in the reference/alternative allele.

6 Deletion compared with reference.

7 Insertion compared with reference.

* Significant association between coagulation trait and AF (*P* < 0.05).

Half the new SNPs could be found in the *CSN1S1* gene. Both rs137349211C>T and rs379988962G>T on intron 13 in *CSN1S1* and intron 5 in *CSN1S2* are situated in the splice polypyrimidine tract. On introns 1, 2, 4, 13, and 14 in *CSN1S1* (Table 2) there are deletions that have not previously been reported whereof 3 are situated in the splice polypyrimidine tract of the intron. Furthermore, the new SNPs ss9732325462, ss9732325455, ss9732325458, and ss9732325457 coincide with protein variant *CSN1S1*^C^. The cows having *CSN1S1*^C^ differ from the reference sequence in 79 of 117 SNPs and seem to form a very clear haplotype.

The potential significance of the SNPs found in this study was investigated through a straightforward chi-squared test and through comparisons with previous GWAS studies. Grouping the cows with regard to the rennet coagulation properties of their milk, a chi-squared test could give indications of any possible association between individual SNPs and coagulation properties. Twelve SNPs were found to be associated with coagulation properties at a nominal significance level of *P* < 0.05; 8 of these SNPs are presented in Table 1, Table 2 together with the allele frequency for the group of cows (n = 13) giving NC milk. The SNP rs109900747, presented in Table 1, was found to be associated with coagulation (*P* < 0.03), where the frequency of the C allele was higher than the expected frequency in the cows giving NC milk. It is plausible that the suggested SNP variation (Vanvanhossou et al., 2021) could have an effect on α_S2_-CN expression, which in turn could affect coagulation properties. Furthermore, the chi-squared analysis showed that SNPs rs110808655, rs133836703, and rs110466181 (Table 2) were associated with coagulation (*P* < 0.03) and that the frequency of the T allele was higher than expected in NC cows. Also, the SNPs rs110291532 and rs136419748 (Table 2) were found to be associated with milk coagulation (*P* < 0.03) where the insertion of A (rs110291532) and the T allele (rs13641974) are more frequent in NC cows. Because the chi-squared test was performed on a large number of SNPs and a small number of animals, these results should be seen as an indication and need to be confirmed in a larger sample set. We have found that SNPs found in this study have been shown to be significant for protein composition and rennet coagulation traits in several GWAS studies. Buitenhuis et al. (2016), who performed a GWAS on milk from Danish Holstein, found that the SNPs rs110808655 and rs110122319 were associated with the relative concentration of α_S2_-CN. Furthermore, rs110808655 has been shown to be associated with the concentration of β-CN in another study (Huang et al., 2012). Pausch et al. (2017) and Sanchez et al. (2017) found that the SNP rs109193501 was associated with total protein and α_S2_-CN concentration, respectively. Pegolo et al. (2018) found that rs110440863 was associated with relative concentrations of κ- and β-CN. Interestingly, the same SNP has also been shown to be associated with rennet coagulation time (Dadousis et al., 2017) and curd firmness (Dadousis et al., 2016). This suggests that some of the SNPs in this small subset could be potential markers for protein composition and rennet coagulation. It must be noted that the chi-squared analysis presented here is only a preliminary analysis with limited statistical power that also cannot account for the variation between the different farms. We will do an in-depth study of the phenotypes by proteomic studies of the resulting CN and their post-translational modifications using LC-HRMS, which will be published in a later stage.

In a small sample set of 30 cows with divergent phenotypes, genetic variation was shown in the CN genes and the variation predominantly occurs in the introns. Genetic variation in the exons confirmed the genetic protein variants of the CN that we have previously reported from analyzing the phenotypic variation in the milk from the same animals. The genetic variants in the 5′- and 3′-UTR in *CSN1S1* and *CSN3*, rs110899610T and rs109787476T, are found together with *CSN1S1*^C^ and *CSN3*^B^, respectively. Because both these variants are associated with gain and loss of different miRNA and TF, this could explain differences in expression of the genetic protein variants. Chi-squared analysis as well as comparisons with previous GWAS studies show potential connections between the identified SNPs and coagulation properties of milk. Further research will focus on phenotypic variation in the milk protein sequences with investigations of detailed variation in post-translational modifications.
